# Supplementary material for: The comparative effectiveness of progressive relaxation training on pain characteristics, attack frequency, activity self-efficacy, and pain-related disability in women with episodic tension-type headache and migraine
Source: PLoS One. 2025 Apr 28;20(4):e0320575. doi: 10.1371/journal.pone.0320575 (PMC12036860; doi:10.1371/journal.pone.0320575)
Supplement: S2 File — (DOCX) [file pone.0320575.s002.docx]

**STUDY PROTOCOL**

| **Title of the Study** | Investigation of the Effect of Progressive Relaxation Training on Pain Characteristics, Attack Frequency, Activity Self-Efficacy Perception and Pain-Related Disability in Women with Tension Type Headache and  Migraine |
| --- | --- |

| **Type of the** |  | Observational Drug Study |
| --- | --- | --- |
| **Research** |  |  |
|  |  | Medical Device Clinical Researc |
|  |  |  |
|  |  | Performance Evaluation Studies |
|  |  | Conducted with in Vitro Medical Diagnostic Devices |
|  |  | Non-pharmaceutical Clinical Resear |
|  | X | Please specify if other: **Prospective/Single Center** |

| **Research Topic** | Headache is a very common health problem in the society (1). It causes limitation of physical functionality of individuals, labor and economic losses, negative effects on sleep quality and poor quality of life (1, 2). Headache types are categorized in two main groups as primary and secondary headaches (3). Headaches are classified as “primary headache” when an organic cause cannot be determined and as “secondary headache” when they are related with an organic pathology such as trauma, infection or tumor (3). According to the International Headache Society (IHS), nearly 90% of headaches are primary headaches and tension-type headaches (TTH) and migraine are the most important examples (3). TTH is felt as heaviness, burning, pressure and squeezing in the head, mild to moderate in intensity and bilaterally localized (3). Pain does not start with climbing stairs or similar activities. There is no nausea or vomiting. Photophobia or phonophobia is absent or only one is present. There may be a sensitive point on one side of the head on the back or neck on which pain increases when pressed (4,5). According to IHS criteria, the prevalence of women diagnosed with TTH is quite high (3,4). In a study with 2226 samples, 453 women experienced tension-type headache and the prevalence was determined as 22.71% for women (4). The prevalence of TTH reaches its maximum level at the ages of 20-50 (5,6). TTH is divided into episodic and chronic types according to pain types. It is called chronic if it lasts longer than 15 days in a month and episodic type if it lasts less than 15 days (7). The clinical history may include a stressful event related to the patient's private life, excessive fatigue, insomnia, excessive physical activity and cold/humid weather (8-10). Factors that decrease TTH include warm and dry air, taking a hot shower, good sleep quality, exercise and massage (11,12). The duration of pain lasts between half an hour and up to 72 hours and is recurrent (6). It has been reported that recurrent TTH attacks may negatively affect the quality of life, family and work life of women (13). In a study conducted in 1573 young individuals with headache in Canada, it was reported that young people diagnosed with TTH canceled activities related to family, work and social life due to pain (13).  Migraine is a multifactorial neurovascular syndrome characterized by moderate to severe headache attacks that occur with triggering factors in women with genetic susceptibility, are usually unilateral and/or throbbing, can be triggered by routine physical activity, and are accompanied by nausea, vomiting, photophobia and phonophobia (3,11). In some patients, attacks may be accompanied by aura with focal neurologic symptoms (11,12). Migraine, which affects more than 10% of the general population, negatively affects the quality of life of young adults by limiting their social life, negatively affecting their professional life, causing loss of labor force, negatively affecting their physical and emotional functions and causing disability (9-13). It is more common in individuals with high intellectual level and 'perfectionists' (14-16). The pain lasts between 4-72 hours (14-16). Various studies on migraine have reported that more than half of the individuals with migraine experience severe headache lasting between 4-24 hours (4,17). In another study conducted in Germany, it was reported that 66% of individuals with migraine had a history of one or two attacks per month, the mean number of attacks was 2.82 days per month and the pain level was moderate in 36% of women and severe in more than 60% (17).It has  also been reported that women with migraine have problems in the management |
| --- | --- |

of housework and participation in social and family activities (18,19).

In the literature, there are various differences in the mechanism of occurrence of TTH and Migraine and the populations affected (10,16-18). It has been found that the lifetime prevalence of TTH is between 34.8% and 78% and the prevalence in women is between 37.1% and 88% (10,16-18). Migraine is 3 times more common in women than in men (4-8). When the pathophysiology of TTH is examined, many studies have emphasized the presence of abnormal metabolism in peripheral muscles (inflammatory reaction, decreased blood flow, increased muscle activity and muscle atrophy) as the basic mechanism (19,20). Increased tenderness in pericranial myofascial structures due to palpation is the most characteristic clinical finding of TTH and this is positively correlated with the frequency and severity of headache (19). It is also thought that sensitivity in the cranial muscles in TTH may occur as a result of psychological or physical stress. Considering the prevalence, it is emphasized that the relation of TTH with ovarian hormones is not clear (21).

Migraine and THH has been observed to reach the highest prevalence in women between the ages of 25-55 (21,22). Therefore, it is emphasized that migraine in women may be related with ovarian hormones (21). It is thought that migraine occurs as a result of increased sensitivity of dopamine receptors and changes in the serotonergic transmission mechanism as a result of changes in estrogen levels with the menstrual cycle (hormonal changes, menstrual cycle and pregnancy) (21,22).Decreases or sudden decreases in estrogen levels increase pain sensitivity. It has been reported that especially migraine-type headache occurs together with neck, back and other joint pains (22,23). In the study conducted by Eroğlu et al. was observed that head and neck pain was more common in menstruating patients because estrogen level decreased during menstrual period (23,24). The increase in nociception seen in the muscles due to this sensitivity may trigger an attack in the person by changing the pain modulation (23,24). Therefore, the processes related to migraine and tension-type headache in women should be examined (26,27).

For individuals, participation in purposeful activities is an essential part of health and well-being (28,29). When people engage in work, play and activities of daily living, they maintain, strengthen, shape and change their capacities, beliefs and dispositions (28,29). Activity self-efficacy includes a sense of personal competence and satisfaction about the activities that individuals perform (28,29). High self-efficacy may affect the sustainability of physical activity and productivity of individuals (29-31). Migraine and TTHA are observed between the ages of 25-55, which is the age when people are most productive and efficient (4-6). It has also been reported that quality of life and well- being are affected in individuals with migraine and TTH, with 31%-51% of individuals taking time off work and decreased work performance (30-32).

Moreover, it is known that TTH and migraine not only cause headache in women but also affect muscle groups used in activities of daily living (19,23). It has also been found that patients have significant deficiencies in functional activity levels (31). In this context, pain, which is a subjective perception, is likely to change activity self- efficacy and perception in women (30-32). It has been reported that the perception of activity changes in women with fibromyalgia, and individuals' self-efficacy and sense of activity satisfaction change (33). Occupational therapy affects coping and self- management skills of women with limitations in participation in activities of daily living (30). In this sense, it is important to examine how TTH and migraine, which have

important social and individual effects, change the perception of activity in individuals.Occupational therapy aims to maximize the physical and psychological performance of women and groups through participation in daily activities that are meaningful for them and to increase social participation skills (29,30,34). Constantly thinking about pain and avoiding physical activity may lead to loss of function in individuals (35). Due to the personalized and complex structure of human roles, each individual diagnosed with headache is likely to encounter different limitations in terms of fulfilling daily roles and social participation, depending on lifestyle and preference (35,36). In this context, there is a need for studies examining social participation in women with TTH and Migraine. When we examined the literature, it was reported that women with migraine and TTH had significant deficiencies in activities of daily living and physical functions, impaired sleep quality and posture disorders (37-40).In this context, nonpharmacologic treatment methods (physiotherapy applications, electromyographic biofeedback, aerobic exercises, posture training, cognitive therapy, acupuncture, yoga, meditation, T'ai-Chi, body awareness therapy and progressive relaxation training) that provide relaxation of various muscle groups are applied in women with TTH and migraine (40-42). One of these treatment methods is progressive muscle relaxation training (39).

Progressive muscle relaxation training is defined as a method that provides relaxation in the whole body by voluntary, regular relaxation of large muscle groups in the human body (39). It has been found that relaxation training has many benefits such as reducing muscle tension, the effects of stress, anxiety, depression, blood pressure, sensitivity to pain and fatigue, facilitating transition to sleep and improving quality of life (39-42). Relaxation especially for the muscle groups (hands, arms, neck, shoulders, face, chest, abdomen, hips, feet and fingers) that are most commonly used in daily living activities increases the benefits for individuals (39). In a study by occupational therapists, it was determined that progressive relaxation training in addition to pain management in individuals with chronic pain (individuals with chronic musculosketal pain, fibromyalgia, chronic headache and migraine) increased their occupational performance, participation in activities of daily living and quality of life (43,44). However, there is a need to examine the effect of progressive relaxation training on pain characteristics, frequency of attacks, perception of activity self-efficacy and pain- related disability İN women with TTH and migraine. With this study, it is thought that it will be beneficial for occupational therapists working with women with migraine and tension-type headache in the clinic, will allow a more holistic evaluation of individuals, and will shed light on more comprehensive intervention approaches against the problems they face in daily life.

The aim of this study is to Investigation of the Effect of Progressive Relaxation Training on Pain Characteristics, Attack Frequency, Activity Self-Efficacy Perception and Pain-Related Disability in Women with Tension Type Headache and Migraine.

| **Sample Profile** | The research population is women with tension-type headache and migraine. The research sample is women studying and working at Çankırı Karatekin University, Faculty of Health Sciences, Department of Occupational Therapy. The research group will consist of women who continue to participate in the study. The women participating in the study will be divided into 2 groups as those with tension-type headache and migraine.  Power analysis was applied to determine the sample size of the study.The G* Power software (G* Power, Version 3.1.9.7 Franz Faul, Universität Kiel, Germany) was used to determine the sample size. The preliminary hypothesis was defined as difference in VAS scores over time (pre-test and post-test) and between groups. Accordingly, it was assumed that the time and group effects would have a moderate effect size in our study. With a Type I error rate of 𝛼=0.05 α=0.05 (95% confidence level) and a desired power of 1−𝛽=0.80 1−β=0.80, the required sample size for statistical analyses was calculated as 20 (n=20).  **Inclusion criteria:**  -  be between the ages of 20-45  -Being diagnosed with Migraine and TTH according to the International Classification of Headache Disorders (ICHD-II) (3)  -Volunteering to participate in the study  **Exclusion criteria of the study;**   - Having disc herniation, radiculopathy, surgical history, tumor, cyst or similar findings in the cervical region   -Having received any physical therapy for the cervical region within 3 months  -Having any psychological disorder   - Being pregnant   -Being in menopause  -When 15% of the trainings are not attended   - Having a mental illness (diagnosed with depression, taking antidepressant medication)   -Having a chronic, neurological or rheumatic condition  -Having sinusitis   - Continuous use of migraine prophylaxis medications   -Not volunteering to participate in the study  Information about the purpose and content of the study will be given. A consent  form will be obtained from the women who voluntarily participate in the study. Progressive relaxation training will be given to both groups |
| --- | --- |

| **Data Collection Methods and Tools** | Women will be divided into two groups as TTH and Migraine group. Progressive relaxation training will be given to both groups. It is planned to evaluate and compare the varying headache severity, headache- related symptoms, number of attacks, activity self-efficacy perceptions and social participation of both groups according to their gender and the type of headache they have before and after the training with evaluation forms.  Sociodemographic characteristics of the women will be evaluated with the demographic information form, pain intensity and number of attacks of women with migraine and TTH will be evaluated with the Visual Analog Scale (VAS), headache severity will be evaluated with the Headache Impact Test (HIT-6), activity self-efficacy perception will be evaluated with the Activity Self-Assessment Scale (OSA), and social participation will be evaluated with the Pain-Related Disability Assessment Schedule (World Health Organization Disability Assessment Schedule 2 (WHO-DAS 2).   1. **Demographic information:** In the demographic information section, the demographic age, marital status (“married” and “single”), employment status (“working” and “not working”) and occupation of the individuals participating in the study will be questioned. Educational status will be recorded both numerically (total years of education) and categorically as associate, undergraduate and graduate. In the physical information section, individuals' physical height (m) and body weight (kg) will be recorded. Current body mass index (BMI) values will be calculated in kg /m2 by dividing current body weight (kg) by the square of current height (m). Their diagnosis according to IHS will be recorded as TTH and Migraine. Duration and frequency of number of attacks in seconds, minutes, hours, will be evaluated in days, weeks, months, years. 2. **Visual analog scale (VAS):** The VAS is one of the most widely used pain measurement tools in researches due to its ease of use and simple structure (45,46). The VAS consists of a 100 millimeter vertical line. At the bottom end of the line is a value of 0, meaning 'no pain', while at the top is a value of 10, meaning 'very severe pain'. The patient is asked to indicate the level of pain on this line. VAS is a test that has been used for a very long time and is accepted in the world literature (46,47) 3. **Headache impact test (HIT-6- Headache Impact Test-6):** The HIT-6 will be used to question individuals about their headache symptoms. It was developed by Gadnek et al. Turkish validity and reliability study was conducted by Dikmen et al. The internal consistency Cronbach a values of   the HIT-6 scores were 0.753 (acceptable) and 0.864 (excellent), |
| --- | --- |

respectively (5). This test, which consists of 6 items including the frequency of headache, the degree of restriction in activities of daily living and social environment due to headache, fatigue and mood changes due to headache, evaluates the problems related to headache in a broad framework. Based on the patient's self-report, it provides quantitative information about migraine and headache. This scale can score between 36 and 78 points. The answers to the test are never, rarely, sometimes, very often, always and the scores for these statements are as follows: Never = 6 points, rarely = 8 points, sometimes = 10 points, very often = 11 points, always = 13 points. It is as follows. This scale can be scored between 36 and 78 points. Grade 1: No impact if the scale score is □ 49, Grade 2: Moderate impact if the scale score is 50-55, Grade 3: 56-59 is considered as significant impairment and grade 4: □ 60 is considered as severe impairment (48,49).

1. **Occupational Self-Assessment Scale (OSAS);** The OSAS is based on the Model of Human Activity (MOHO) and is used to assess the person's activity adequacy and activity worthiness from his/her own perspective (51). The internal consistency Cronbach □ values of the scores of the OSA scale were determined as 0.95-0.96 (excellent), respectively. It is a scale consisting of twenty-one questions and three sections. It includes statements about oneself. Each section consists of two subsections: In the first one, the person's During the first part, the level of difficulty is determined. In the second part, the extent to which these activities is determined to be important. The individual is asked to rate each item on two separate 4-point Likert scales; the first rating is for competence in the activity (1 point= I have a lot of trouble doing this; 2 points= I have difficulty doing this; 3 points= I do this well; and 4 points= I do this extremely well). The second rating is for the value given to the activity (1 point= This is not important to me; 2 points= This is important to me; 3 points= This is more important to me; and 4 points= This is the most important to me). The first 11 items of the test assess skills, questions 12 through 16 assess habits, and questions 17 through 21 assess perception of one's aspirations. A high score indicates a high perception and a low score indicates a low perception. Kielhofner et al. reported that the OSA validly and meaningfully assessed 90% of people with disabilities in various contexts. It was developed by Baron et al. and Pekçetin et al. In

2018, Pekçetin et al. conducted a Turkish validity and reliability study on its use in adult individuals (50,51).

1. **Social participation Disability Assessment Schedule (World Health Organization Disability Assessment Schedule 2, WHO-DAS II):** The WHO-DAS II scale will be used to assess social participation in the study WHO-DAS II was developed by the World Health Organization. It was translated into Turkish by Ulug et al. in 2001, and the validity and reliability study was completed by obtaining Cronbach's alpha coefficients (values between 0.60 and 0.90) for all sub-domains (53,54). The WHO-DAS II is a 36-item semi-structured interview scale. It was developed to determine the limitations in the activity level and social participation of the individual regardless of the medical diagnosis. The test consists of 6 domains including activities that are considered important in many cultures. These are; first domain (DAS 1), understanding and relating; this domain refers to the cognitive processing of the environment. Domain two (DAS 2), moving and getting from one place to another; this domain refers to severe limitation in mobility. The third domain (DAS 3), self-care, includes statements such as bathing, feeding, dressing and being able to be alone. The fourth domain (DAS 4) refers to human relations, being able to enter a social environment, relationships with people close or unfamiliar. Domain five (DAS 5) refers to life activities, including activities related to work, education and housework. Domain six (DAS 6) includes comprehensive questions about participation in social life. In questions related to all these domains, the extent to which the person has had difficulty during that activity in the last month is scored on a scale of 1-5, with answers ranging from not at all, slightly, moderately, very much, extremely/not at all. This assessment takes approximately 20 minutes. During the scoring of WHO-DAS II, domain scores are weighted according to the number of questions and domain scores and total DAS score are evaluated over 100 (52,53).
2. **Pain Catastrophizing Scale (PCS):** Catastrophizing pain was evaluated with the Pain Catastrophizing Scale (PCS). It was developed by Sullivan et al. in 1995, and Turkish validity and reliability study was performed by İlçın N. et al. (55,56). The internal consistency Cronbach □ values of the AFQ scores were determined as 0.95-0.96 (excellent), respectively.

Developed to identify patients' catastrophic thoughts or feelings about the pain they experience and their ineffective coping strategies, the AAS is a Likert-type self-assessment scale consisting of thirteen items. Each item is evaluated between 0-4 points. The total score ranges from 0 to 52. It includes rumination, magnification and helplessness subscales. Three subscores for pain magnification (items 6, 7, and 13), rumination (items 8 to 11), and helplessness (items 1 to 5 and 12) were calculated in addition to the total AFC score. High scores indicate a high level of catastrophizing (54,55).

1. **Progressive Muscle Relaxation Training (PCRT):** Progressive muscle relaxation training is an easy-to-learn, non-invasive and side effect-free method (39). This training will be applied for 6 weeks. It will be planned for individuals 2 times a week (once a day) for a total of 12 sessions. The practice time will be at least 30 minutes for beginners and 15 minutes for practitioners who have learned the basics of the method time will be given.
2. Prior to the application, individuals will be informed that they should not come overly hungry or overly full (eating should be stopped 2 hours before), wear loose and comfortable clothing, avoid clothing that tightly hugs the abdomen (pants, dresses, belts), remove contact lenses, glasses, jewelry and tight shoes. The PCI will be administered in a quiet, comfortable and dimly lit place. Individuals will perform PCI in the supine position. Individuals will be taught deep diaphragmatic breathing before the application. During diaphragmatic breathing, individuals will take deep breaths to activate the diaphragm (preventing the movement of the rib cage during inspiration - ensuring the movement of the upper abdomen). After diaphragm breathing is taught, respectively (56);
   - First of all, training will be started with slow, deep and rhythmic diaphragmatic breathing, which is taken through the nose and given through the mouth.
   - After repeating slow and deep breathing movements several times, progressive muscle relaxation training will be started.
   - In progressive muscle relaxation training, the right foot, left foot, right leg, left leg, buttocks, abdominal muscles, chest muscles and back muscles, right hand, left hand, right arm, left arm, neck and shoulders, facial muscles (all major muscles from bottom to top)

are contracted during deep and slow inhalations and relaxed and released during exhalations. Muscle contraction will be done for 10 seconds and relaxation for 20 seconds.

- After each muscle contraction and relaxation, slow and deep diaphragmatic breaths will be added a few times before moving on to a new muscle group.

9. - After all muscles are contracted and relaxed respectively, this time all muscles are contracted simultaneously while inhaling and relaxed simultaneously while exhaling. Then slow and deep The training is completed with diaphragm breaths (56).

| **Data Collection Process** | It will be held at Çankırı Karatekin University, Faculty of Health Sciences, Department of Occupational Therapy. Individuals suitable for study will be invited to through advertisements on verbal and social media tools. Verbal and written consent will be  obtained from women. |
| --- | --- |
| **Data Analysis and Evaluation Method** | Data analysis will be done with SPSS 24.0 program. The suitability of numerical variables for normal distribution will be evaluated with Kolmogorov-Smirnov. Descriptive analyzes will be given as mean±standard deviation or percentage (%) for variables with normal distribution. Descriptive analyzes will be expressed as median and interquartile range (IQR) for variables with non-normal distribution. In comparing two numerical variables belonging to independent data groups that are not normally distributed, evaluation will be made with the Mann Whitney U test. The Kruskall Wallis test will be used to compare more than 2 numerical data belonging to independent data groups that are not normally distributed. Chi square test -Fisher exact test will be used in cross table analysis. Situations where the P value is  below 0.05 will be considered statistically significant. |

| **REFERENCES** | |
| --- | --- |
| **Queue** | **References** |
| 1. | Steiner, TJ., Stovner, LJ., Katsarava, Z., et al. The impact of headache in Europe: Principal results of theEurolight pro-ject. J Headache Pain 2014; 15: 31. |
| 2. | Linet, M.S., Stewart, W.F., Celentano, D.D., Ziegler, D., Sprecher, M. An epidemiologic study of headache adolescents and young adults. JAMA, 1989,261, 2211-2216, |
| 3. | Headache Classifcation Commitee of the International Headache Society. The international classifcation of headache disorders. Cephalalgia. 2003; 24: 55-7. |
| 4. | Ertas, M., Baykan, B., Orhan, E.K., Zarifoglu, M., Karli, N., Saip, S., Onal, A.E., & Siva,  A. One-year prevalence and the impact of migraine and tension-type headache in Turkey: a nationwide homebased study in adults Journal of Headache and Pain. 2012; 13, 147–157. |
| 5. | Hogg-Johnson, S., Van Der Velde, G., Carroll, L.J., Holm, L.W., Cassidy, J.D., Guzman, J. ve diğerleri. The Sayfa burden and determinants of neck pain in the general population. European Spine Journal.  2008:17 (1), 39-51. |
| 6. | Mavioğlu, H., Karaca, S., Yılmaz, H., Korkmaz, H., Artuğ, R., Selçuki, D. Demographic and Clinical Profile of Headache Outpatient Clinic Patients, Düşünen Adam. 2000; 13(2):  110-115 |
| 7. | Silberstein SD, Lipton RB. Overview of diagnosis and treatment of migraine. Neurology 1994;44 6-1 |
| 8. | Tanik N, Saçmaci H, Aktürk T. The relationship between exposure to hot/cold weather and the clinical features of headaches in patients with migraine and tension-type headaches. Neurol Res 2020;42:239–43 |
| 9. | Wang J, Huang Q, Li N, Tan G, Chen L, Zhou J. Triggers of migraine and tension-type headache in China: a clinic-based survey. Eur J Neurol. 2013 Apr;20(4):689-96. doi: 10.1111/ene.12039. Epub 2013 Jan 28. PMID: 23356519. |
| 10. | Tai, ML.S., Yet, S.X.E., Lim, T.C. et al. Geographical Differences in Trigger Factors of Tension-Type Headaches and Migraines. Curr Pain Headache Rep 23, 12 (2019). https://doi.org/10.1007/ s11916-019-0760-6 |
| 11. | Rasmussen, Birthe Krogh. "Migraine and tension-type headache in a general population: precipitating factors, female hormones, sleep pattern and relation to lifestyle." Pain 53.1 (1993): 65-72. |
| 12. | Söderberg EI, Carlsso JY, Stener-Victorin E, Dahlöf C. Subjective well-being in patients with chronic tension-type headache: efect of acapuncture, physical training, and relaxation training. Clin J Pain 2011; 27: 448–56 |
| 13. | Siva A. Epidemiology of Headache. Türkiye Clinics 2003;(1):94-7 |
| 14. | Dodick DW, Loder EW, Manack Adams A, Buse DC, Fanning KM, Reed ML, et al. Assessing Barriers to Chronic Migraine Consultation, Diagnosis, and Treatment: Results From the Chronic Migraine Epidemiology and Outcomes (CaMEO) Study.  Headache 2016;56(5):821–34. |
| 15. | Raposio E, Bertozzi N. Trigger Site Inactivation for the Surgical Therapy of Occipital Migraine and Tension-type Headache: Our Experience and Review of the Literature. Plastic Reconstructive Surgery.2019:12:7-11,. |

| 16. | Martin PR (2010) Behavioral management of migraine headache triggers: learning to cope withtriggers. Curr Pain Headache Rep 14:221–227 |
| --- | --- |
| 17. | Rasmussen, B. K., Jensen, R., Schroll, M. and Olesen, J. Epidemiology of headache in a generalpopulation—a prevalence study. JCE. 1991;44 (11), 1147–1157. |
| 18. | Göbel, H., PetersenBraun, M., & Soyka, D. The epidemiology of headache in Germany: a nationwide survey of a representative sample on the basis of the headache classifcation of the International Headache Society. Cephalalgia. 1994; 14(2), 97-106 |
| 19. | Siva A. Epidemiology of headache. Turkish Clinics Neurology Journal 2003; 1:94-97. |
| 20. | France S, Bown J, Nowosilskyj M, et al. Evidence for the use of dry needling and physiotherapy in the management of cervicogenic or tension-type headache: A systematic review. Cephalalgia2014: 0(0), 1-10. |
| 21. | Bendtsen L, Fernandez-de-la-Penas C. The role of muscles in tension-type headache. CurrPain Headache Rep 2011:15(6), 451– 8. |
| 22. | Todd, C., Lagman-Bartolome, A.M. & Lay, C. Women and Migraine: the Role of Hormones. Curr Neurol Neurosci Rep 18, 42 (2018). https://doi.org/10.1007/s11910-018-0845-3 |
| 23. | Todd, C., Lagman-Bartolome, A.M. & Lay, C. Women and Migraine: the Role of Hormones. Curr Neurol Neurosci Rep 18, 42 (2018). https://doi.org/10.1007/s11910-018-0845-3 |
| 24. | Rantala MA, Ahlberg J, Suvinen TI, Nissinen M, Lindholm H, Savolainen A, Könönen M. Temporomandibular joint related painless symptoms, orofacial pain, neck pain, headache, and psychosocial factors among non-patients. Acta Odontol Scand  2003;61(4):217-22. |
| 25. | Eroğlu, C. Neslihan, Keskin Tunç S, and Elesan S. Investigation of the frequency of temporomandibular joint, head-neck, face-ear pain during and before the menstrual period: Pilot study. Atatürk University Faculty of Dentistry Journal 28.2: 157-161., |
| 26. | Burch, Rebecca. "Migraine and tension-type headache: diagnosis and treatment." Medical Clinics 103.2(2019): 215-233. |
| 27. | Probyn, K., Bowers, H., Mistry, D., Caldwell, F., Underwood, M., Patel, S., Sandhu, H.K., Matharu, M. and Pincus, T., 2017. Non-pharmacological self-management for people living with migraine or tension- type headache: a systematic review including analysis of intervention components. BMJ open, 7(8), p.e016670. |
| 28. | Söderberg E, Carlsson J, Stener-Victorin E. Chronic Tension-Type Headache Treated with Acupuncture, Physical Training and Relaxation Training. Between-Group Differences. Cephalalgia.  2006;26(11):1320-1329. doi:10.1111/j.1468-2982.2006.01209.x |
| 29. | DA Marcus; L Scharff; S Mercer; DC Turk (1998). Nonpharmacological treatment for migraine: incremental utility of physical therapy with relaxation and thermal biofeedback. , 18(5), –. doi:10.1111/j.1468-2982.1998.1805266.x |
| 30. | 2Kaplan, SE,Ovayolu, N and Ovayolu Ö. "The effect of progressive relaxation exercises on pain, fatigue, and quality of life in dialysis patients." Holistic Nursing Practice 34.2 (2020): 121-128. |
| 31. | McCallie MS, Blum CM, Hood CJ. Progressive muscle relaxation, Journal of Human Behavior in theSocial Environment, 2006,13(3):51-66 |

| 32. | Samuelsson, Kersti, et al. "Patient-reported outcome of a multidisciplinary pain management program, focusing on occupational performance and satisfaction with performance.The OpenRehabilitation Journal. (2011). |
| --- | --- |
| 33. | Espí-López, Gemma Victoria, et al. "Effectiveness of physical therapy in patients with tension-type headache: literature review." Journal of the Japanese Physical Therapy Association (2014): Vol17_005. |
| 34. | Fichtel, Åsa, and Bo Larsson. "Does relaxation treatment have differential effects on migraine and tension□type headache in adolescents?." Headache: The Journal of Head and Face Pain 41.3 (2001):290-296. |
| 35. | Stovner L. J. Nichols E. Steiner T. J et al. Global, regional, and national burden of migraine and tensiontype headache,: A systematic analysis for the Global Burden of Disease Study 2016. The Lancet Neurology. 2018;17(11), 954–976. 10.1016/S1474-  4422(18)30322-3 |
| 36. | Leonardi, M., Raggi, A., Ajovalasit, D., Bussone, G., & D’Amico, D. Functioning and disability in migraine.Disability and Rehabilitation. 2010;32(S1), S23-32. |
| 37. | Pryse-Phillips W, Findlay H, Tugwell P, Edmeads J, Murray TJ, Nelson RF. A Canadian population survey on the clinical, epidemiologic and societal impact of migraine and tension-type headache. Can J NeurolSci. 1992;19(3):333-339 |
| 38. | Jonsson H, Borell L, Sadlo G. Retirement: An occupational transition with consequences for temporality, balance and meaning of occupations. Journal of Occupational Science. 2000;7(1): 29-37 |
| 39. | Lagueux, Emilie, Andréa Dépelteau, and Julie Masse. "Occupational therapy’s unique contribution to chronic pain management: A scoping review." Pain Research and Management .2018;1-19. |
| 40. | Reshu Gupta1, Prahlad Dhakar2, Jitendra Ahuja3, et al Clinical Characteristics of a Severe Headache and Its Impact on Personal Life of Patients Int J Cur Res Rev. 2021;13( 10)95-100 |
| 41. | McLean A, Coutts K. Occupational therapy: self-management for people with migraines. Occup TherNow. 2011;13:5–8. |
| 42. | Rivano-Fischer M, Eklund M. Evaluation of changes in occupational performance among patients in a pain management program. J Rehabil Med. 2004; 36(2):85–91. [PMID: 15180223] DOI:10.1080/16501970310019142 |
| 43. | Leonardi, M., Raggi, A., Ajovalasit, D., Bussone, G., & D’Amico, D. Functioning and disability in migraine.Disability and Rehabilitation. 2010;32(S1), S23-32. |
| 44. | Bera SC, Khandelwal SK, Sood M, Goyal V. A comparative study of psychiatric comorbidity, quality of life and disability in patients with migraine and tension type headache. Neurol India. 2014;62:516-20 |
| 45. | Silberstein, Stephen D., et al. "The International Classification of Headache Disorders, (ICHD-II)—- Revision of Criteria for 8.2 Medication-Overuse Headache." Cephalalgia 25.6 (2005): 460-465. |
| 46. | Price, D. D., McGrath, P. A., Rafii A., & Buckingham, B. (1983). The validation of visual analogue scalesas ratio scale measures for chronic and experimental pain. Pain, 17, 45-56 |
| 47. | Hawker, G. A., Mian, S., Kendzerska, T., & French, M. (2011). Measures of adult pain: Visual analog scalefor pain (vas pain), numeric rating scale for pain (nrs pain), mcgill pain questionnaire (mpq), short□ form mcgill pain questionnaire (sf□mpq), chronic pain grade scale (cpgs), short form□36 bodily pain scale (sf□36 bps), and measure of intermittent and constant osteoarthritis pain (icoap). Arthritis Care Res, 63(11),  240-252 |

| 48. | Gandek B, Alacoque J, Uzun V, Andrew-Hobbs M, Davis K. Translating the Short-Form Headache Impact Test (HIT-6) in 27 countries: Methodological and conceptual issues. Qual Life Res 2003;12:975–979. [Crossref] |
| --- | --- |
| 49. | Dikmen PY, Bozdağ M, Güneş M, Koşak S, Taşdelen B, Uluduz D, Ozge A. Reliability and validity of Turkish Version of Headache Impact Test (HIT-6) in patients with migraine. Arch Neuropsychiatry 2020. |
| 50. | Kielhofner G, Forsyth K, Kramer J, Iyenger A. Developing the Occupational Self Assessment: the use of Rasch analysis to assure internal validity, sensitivity and reliability. British Journal of Occupational Therapy. 2009;72(3):94-104. 102. |
| 51. | Pekçetin S, Salar S, İnal Ö, Kayıhan H. Validity of the Turkish Occupational Self Assessment for Elderly Individuals. OTJR: occupation, participation and health. 2018;38(2):105-12. |
| 52. | World Health Organisation (1999) WHO Disability Assessment Schedule-II (WHO-DAS-II  ) |
| 53. | Uluğ B., Ertuğrul A., Göğüş A., Kabakçı E. (2001) Validity and Reliability of the Disability Assessment Schedule in Schizophrenia Patients. Turkish Journal of Psychiatry, 12(2):121-130. |
| 54. | Sullivan MJL, Bishop SC, Pivik J. The pain catastrophizing scale: development and validation. Psychol Assess 1995; 7:524-532 56. Süren M, Okan I, Go□ kbakanAM, Kaya Z, Erkorkmaz U□ A, rici S, et al. Factors associated with the pain catastrophizing scale and validation in a sample of the Turkish population. Turkish journal of  medical sciences. 2014;44(1):104-107 |
| 55. | Süren M, Okan I, Gökbakan AM, Kaya Z, Erkorkmaz U, Arici S, Karaman S, Kahveci M. Factors associated with the pain catastrophizing scale and validation in a sample of the Turkish population. Turk J Med Sci. 2014;44(1):104-8. doi: 10.3906/sag-1206-67.  PMID: 25558568. |
| 56. | Kwekkeboom KL, Hau H, Wanta B, Bumpus M. Patients’ perceptions of the effectiveness of guied imagery and progressive muscle relaxation interventions used for cancer pain. Complement Ther ClinPract 2008; 14(19): 185-194. |
