# Supplementary material for: The comparative effectiveness of progressive relaxation training on pain characteristics, attack frequency, activity self-efficacy, and pain-related disability in women with episodic tension-type headache and migraine
Source: PLoS One. 2025 Apr 28;20(4):e0320575. doi: 10.1371/journal.pone.0320575 (PMC12036860; doi:10.1371/journal.pone.0320575)
Supplement: Attachment — Submitted filename: 24.01.2025 Plos One Response to Reviewer .docx [file pone.0320575.s005.docx]

PONE-D-24-36994R1

The Comparative Effectiveness of Progressive Relaxation Training on Pain Characteristics, Attack Frequency, Activity Self-Efficacy, and Pain-Related Disability in Women with Episodic Tension-Type Headache and Migraine

PLOS ONE
**Comments to the Author**

5. Is the manuscript presented in an intelligible fashion and written in standard English?

PLOS ONE does not copyedit accepted manuscripts, so the language in submitted articles must be clear, correct, and unambiguous. Any typographical or grammatical errors should be corrected at revision, so please note any specific errors here.

Reviewer #2: Yes

Reviewer #3: No

Reviewer #4: No

Reviewer #5: Yes

Thank you for your comment. The manuscript has been thoroughly reviewed and revised to ensure it is presented in an intelligible fashion and written in standard English.

The study was statistically and methodologically reviewed.
Please make the following corrections:

The article is a clinical trial exploring the effectiveness of progressive relaxation training (PRT) in women with episodic tension-type headache (TTH) and migraine. Below are the identified flaws and areas for improvement:

The lack of evaluator blinding introduces bias, especially in subjective assessments such as pain scales.

Thank you for your comment. Yes, you are right. To minimize bias in the study, the statistician was blinded. In the literature, most studies conducted on conditions such as migraine and tension-type headaches have used the VAS (Visual Analogue Scale) (R1, R2). Therefore, we also used a subjective assessment method and women were asked to mark the intensity of their headaches on the horizontal lines and the results were recorded in centimeters. Additionally, the evaluators ensured that the women completed the forms entirely based on self-reported pain scales. However, the lack of blinding for the evaluators and participants regarding the study group represents a limitation. This has been addressed in the limitations section of the manuscript.

R1: Peng, K. P., & Wang, S. J. (2012). Migraine diagnosis: screening items, instruments, and scales. *Acta Anaesthesiologica Taiwanica*, *50*(2), 69-73.

R2: Houts, C. R., McGinley, J. S., Nishida, T. K., Buse, D. C., Wirth, R. J., Dodick, D. W., ... & Lipton, R. B. (2021). Systematic review of outcomes and endpoints in acute migraine clinical trials. *Headache: The Journal of Head and Face Pain*, *61*(2), 263-275.

The study does not include a placebo or alternative intervention group, which limits the robustness of the conclusions regarding the specific effects of PRT.

Yes, you are right. In the study, most of the women with migraine and tension-type headache tend to start medication use in the early stages. The literature also indicates that medication use begins early in cases of severe pain (R1,R2). For this reason, obtaining the desired control group was challenging. Nevertheless, the absence of placebo and control groups has been included as a limitation.

R1: Bigal, M. E., & Lipton, R. B. (2009). Overuse of acute migraine medications and migraine chronification. Current pain and headache reports, 13, 301-307.

R2: Zwart, J. A., Dyb, G., Hagen, K. M. D. P., Svebak, S., & Holmen, J. M. D. P. (2003). Analgesic use: a predictor of chronic pain and medication overuse headache: the head–HUNT study. Neurology, 61(2), 160-164.

While a post-hoc analysis showed sufficient power, the small sample size (n=20 per group) may affect the generalizability of results and could lead to an overestimation of effect sizes. Median summaries are not consistently provided despite the small sample size, which might skew the interpretation of results.

Yes you are right. Post-hoc power analysis was conducted after the study to evaluate whether the sample size used was sufficient. The post-hoc power analysis revealed that the power of the study was 98%. This indicates that the sample size used in the study was statistically sufficient. Therefore, the Statistical Analysis section has been revised to include additional details (between lines 229-237). Median scores and delta scores have been expanded by adding Table (1),(2),(3) and (4) (between lines 302-330).Thank you for your feedback.

Added text in Statistical analysis Section: The G* Power software (G* Power, Version 3.1.9.7 Franz Faul, Universität Kiel, Germany) was used to determine the sample size. The preliminary hypothesis was defined as difference in VAS scores over time (pre-test and post-test) and between groups. Accordingly, it was assumed that the time and group effects would have a moderate effect size in our study. With a Type I error rate of 𝛼=0.05 α=0.05 (95% confidence level) and a desired power of 1−𝛽=0.80 1−β=0.80, the required sample size for statistical analyses was calculated as 20 (n=20) (R1).

Post-hoc power analysis was conducted after the study to evaluate whether the sample size used was sufficient.

The post-hoc power analysis revealed that the power of the study was 98%. This indicates that the sample size used in the study was statistically sufficient.

R1: Faul F, Erdfelder E, Buchner A, Lang AG. 2009. Statistical power analyses using G* Power 3.1: Tests for correlation and regression analyses. Behavior Res Met, 41: 1149-1160)

The paper does not mention adjustments for multiple statistical tests, potentially inflating the risk of Type I errors. Limited details about multivariate analysis or rationale for specific tests reduce the clarity of the statistical approach.

Thank your for comment. The Statistical Analysis section has been revised to include additional details (between lines 308-237)**.** Median scores and delta scores have been expanded by adding Table 4 (between lines 302-330).

Added text in *Statistical analysis Section*: The G* Power software (G* Power, Version 3.1.9.7 Franz Faul, Universität Kiel, Germany) was used to determine the sample size. The preliminary hypothesis was defined as difference in VAS scores over time (pre-test and post-test) and between groups. Accordingly, it was assumed that the time and group effects would have a moderate effect size in our study. With a Type I error rate of 𝛼=0.05 α=0.05 (95% confidence level) and a desired power of 1−𝛽=0.80 1−β=0.80, the required sample size for statistical analyses was calculated as 20 (n=20) (R1).. If you have any other corrections or modifications you would like to request, we are open to making them.

R1: Faul F, Erdfelder E, Buchner A, Lang AG. 2009. Statistical power analyses using G* Power 3.1: Tests for correlation and regression analyses. Behavior Res Met, 41: 1149-1160)

Inclusion of only women within a narrow age range (20–45 years) limits the applicability of findings to broader populations, including men and older individuals. The inclusion criteria (recent diagnoses for migraines and TTH) might not reflect chronic headache sufferers, a significant subgroup in clinical practice.

Thank you for your valuable feedback. As mentioned in the literature, migraine and TTH are predominantly seen in women wtihin narrow age range, which guided our focus on this population (R3,R4). Additionally, individuals with chronic migraine are likely to have more established health-seeking behaviors and medication use compared to those with episodic migraine (R5,R6). These factors may influence both their treatment outcomes and their participation in non-pharmacological interventions like Progressive Relaxation Training . While our study specifically targeted women with episodic headaches, we agree that future research should explore the effects of PRT in individuals with chronic migraine and TTH, as well as in broader and more diverse populations, to enhance the generalizability of the findings. We have included this in the discussion and recommendations for future studies. However, if you have a further suggestion, we will revise the manuscript in accordance with your suggestion.

R3: Smitherman, T. A., Burch, R., Sheikh, H., & Loder, E. (2013). The Prevalence, Impact, and Treatment of Migraine and Severe Headaches in the U nited S tates: A Review of Statistics From National Surveillance Studies. Headache: The Journal of Head and Face Pain, 53(3), 427-436.

R4:Neumeier, M. S., Pohl, H., Sandor, P. S., Gut, H., Merki-Feld, G. S., & Andrée, C. (2021). Dealing with headache: sex differences in the burden of migraine-and tension-type headache. Brain sciences, 11(10), 1323.

R5: Seng, E. K., Martin, P. R., & Houle, T. T. (2022). Lifestyle factors and migraine. The Lancet Neurology, 21(10), 911-921.

R6: Hagen, K., Åsberg, A. N., Stovner, L., Linde, M., Zwart, J. A., Winsvold, B. S., & Heuch, I. (2018). Lifestyle factors and risk of migraine and tension-type headache. Follow-up data from the Nord-Trøndelag Health Surveys 1995–1997 and 2006–2008. *Cephalalgia*, *38*(13), 1919-1926.

The rationale for comparing outcomes between two different headache types, which inherently have different pathophysiological mechanisms, is unclear and complicates interpretation.

Response: Thank you for your comments regarding the study design and different pathophysiological mechanisms of headaches. Fichtel and Larsson (2001) conducted a similar study in adolescents with TTH and migraines, applying PRT to two different headache groups (R1). Therefore, in the Study Design section, we have added further clarifications to improve understanding of our design between lines 128-131. Additionally, We also rearranged the title to "The Comparative Effectiveness of Progressive Relaxation Training on Pain Characteristics, Attack Frequency, Activity Self-Efficacy, and Pain-Related Disability in Women with Episodic Tension-Type Headache and Migraine" to match the changes we made to the method. Furthermore, we have revised the sample size (between lines 229-231) in the statistical analysis section for clarity. We hope that these changes will meet your expectations. These changes have also been reflected in the abstract to ensure consistency.

However, if you have a further suggestion, we will revise the manuscript in accordance with your suggestion.

Added Text in Study Design Section: "This study is prospective, experimental between-group study with premeasurements and postmeasurements. In this study, TTH and migraines participated in PRT sessions twice a week for six weeks. The aim of this study was to evaluate and compare the outcomes of these two distinct groups after the training.

The discussion lacks depth in explaining how PRT impacts the distinct mechanisms of migraine and TTH.
Thank you for your comment. The details regarding the pathophysiology of Migraine and TTH, as well as the Progressive Relaxation Training, are provided between lines 382 and 399 in the manuscript. However, we have expanded the discussion to explore the physiological differences between migraine and tension-type headache (TTH) patients. Migraine is often associated with neurovascular mechanisms and heightened central sensitization, whereas TTH is predominantly linked to muscular tension and peripheral pain mechanisms [32,33]. These differences may influence the effectiveness of Progressive Relaxation Training, which targets muscle tension and promotes overall relaxation. Our findings suggest that while both groups benefit from PRT, the mechanisms underlying these benefits may differ. This has been discussed in the manuscript to provide a more comprehensive understanding of how these physiological differences relate to our study’s discussion (between lines 378-381). We hope that these changes will meet your expectations. However, if you have a further suggestion, we will revise the manuscript in accordance with your suggestion.

Added text in Discussion Section: Migraine is often associated with neurovascular mechanisms and heightened central sensitization, whereas TTH is predominantly linked to muscular tension and peripheral pain mechanisms. These differences may influence the effectiveness of PRT, which targets muscle tension and promotes overall relaxation [4,32,33].

Thank you very much for your comments. Revisions have been made based on your suggestions. We are open to making further adjustments if you require.

Uneven distribution of education levels between groups is noted but not adequately discussed regarding its potential impact on outcomes.

Thank you for pointing this out. In our study, women with higher education levels were present in both groups. 95.0% of women in the Migraine group were university graduates, while in the TTH group, 55.0% were university graduates. In the literature, the results regarding the relationship between education level and TTH and migraine are controversial. Some studies associate high education level with migraine and TTH (R5, R6), while in others, the effect of education level is modest (R7,R8,R9). Education reflects an individual's intellectual abilities and may also be associated with different health behaviors (R10). In the study, women had a higher level of education. This may have provided an advantage in seeing the actual effect of the training given to both groups. This point is included in the strengths of the study**.** However, if you have a further suggestion, we will revise the manuscript in accordance with your suggestion.

Added Text in the Discussion-Strengths of the Study**:**‘’In the study, women with higher education levels were present in both groups. 95.0% of women in the Migraine group were university graduates, while in the TTH group, 55.0% were university graduates. Education reflects an individual's intellectual abilities and may also be associated with different health behaviors (47). Both women with TTH and migraine were a high level of education. It may have been advantageous in enabling patients to better understand and apply the PRT and thus reveal the true effect of the PRT in both groups (47).

R5. Yang, H., Pu, S., Yang, L., Luo, W., Zhao, J., Liu, E., … & Luo, J. (2022). Migraine among students of a medical college in western china: a cross-sectional study. European Journal of Medical Research, 27(1). https://doi.org/10.1186/s40001-022-00698-9

R6. Vuković, V., Plavec, D., Pavelin, S., Jančuljak, D., Ivanković, M., & Demarin, V. (2010). Prevalence of migraine, probable migraine and tension-type headache in the croatian population. Neuroepidemiology, 35(1), 59-65. https://doi.org/10.1159/000310940

R7. Ertaş, M., Baykan, B., Orhan, E., Zarifoğlu, M., Karlı, N., Saıp, S., … & Síva, A. (2012). One-year prevalence and the impact of migraine and tension-type headache in turkey: a nationwide home-based study in adults. The Journal of Headache and Pain, 13(2), 147-157. https://doi.org/10.1007/s10194-011-0414-5

R8. Molarius A, Tegelberg A, Ohrvik J. Socio-economic factors, lifestyle, and headache disorders - a population-based study in Sweden. Headache. 2008 Nov-Dec;48(10):1426-37. doi: 10.1111/j.1526-4610.2008.01178.x. Epub 2008 Jul 1. PMID: 18624712.

R9. Han, L., Tfelt‐Hansen, P., Skytthe, A., Kyvik, K., & Olesen, J. (2011). Association between migraine, lifestyle and socioeconomic factors: a population-based cross-sectional study. The Journal of Headache and Pain, 12(2), 157-172. https://doi.org/10.1007/s10194-011-0321-9

R10. Skalamera, J. and Hummer, R. (2016). Educational attainment and the clustering of health-related behavior among u.s. young adults. Preventive Medicine, 84, 83-89. https://doi.org/10.1016/j.ypmed.2015.12.011

Although ethical approval is mentioned, additional details about how informed consent was obtained and adherence monitoring are absent.

Thank you for your comments regarding the ethical considerations of our study. We have added a section to the manuscript that outlines the process through which informed consent was secured from all participants. This includes details on the information provided to participants regarding the nature and purpose of the study, potential risks and benefits, and their rights to withdraw at any time without any consequences. Furthermore, we have included additional information on how we monitored adherence to the Progressive Relaxation Training (PRT) throughout the study. This monitoring was conducted through weekly meetings and regular phone calls with the participants. These methods were applied to ensure continuous participation and to address any concerns the participants might have had.

Added Text in the *Participants* Section: Written and verbal consent was obtained by providing information to the women about the nature and purpose of the study, potential risks and benefits, and their rights to withdraw at any time without any consequences. The monitoring process was conducted through weekly meetings and regular phone calls with the participants.

The exclusion of non-adherent participants raises questions about the intervention's feasibility and applicability in real-world settings.

Thank you for your comment. We have optimized the duration of the intervention to maintain participant adherence, acknowledging that prolonged sessions could hinder participation. This approach reflects real-world application, where therapies are often integrated within patient schedules in healthcare settings. Furthermore, these therapies are utilized in patient settings where some institutions implement them as part of workplace health enhancement programs during non-working hours, providing optimal therapeutic service (R1). As mentioned in the study protocol, the study also included women working within the university. This demonstrates that the intervention can be applied even in workplace environments, providing significant evidence of its feasibility in real-world settings. Also, In the study, the women did not drop out due to adverse effects of the treatment or inability to comply with it. Some participants moved to another city, while others were unable to complete the final assessments or maintain the desired level of continuity in the study due to family matters or changes in their health conditions.

R1: Smith, M. (2008). The effects of a single music relaxation session on state anxiety levels of adults in a workplace environment. Australian Journal of Music Therapy, 19, 45-66.

While safety measures are briefly described, a dedicated section detailing adverse effects or participant concerns during PRT sessions is missing.

Thank you for your comment. We ensured the PRT sessions were conducted in a controlled environment equipped with soundproofing, ergonomic seating, privacy measures, accessibility features, emergency preparedness, optimal lighting, and temperature and humidity control to maximize safety and comfort for all participants, and permission was obtained from the selected institution.

Added Text in the *Interventions* Section: Women were instructed to perform the trainings in a safe environment and were advised to stop immediately and report any pain or discomfort they experienced. PRT sessions were conducted in a controlled environment with soundproofing, ergonomic seating, privacy measures, accessibility features, emergency preparedness, optimal lighting, and temperature and humidity control to maximize safety and comfort for all participants.

The inclusion of a CONSORT checklist for a non-RCT study is inappropriate and inconsistent.
Thank you for your comment. The CONSORT checklist was removed from the file.

Reviewer #5: Good paper with enough information, but I did not find all details related to consort checklist. The method section has lots of missing points: like sample size, two group explanation, randomization and blinding process.

Thank you for your comment.

- The CONSORT checklist:

The study is a cohort study. STROBE was added. It was arranged according to the STROBE checklist added.

- The Sample Size:

Thank you for your comments regarding the study design and sample size calculation. Fichtel and Larsson (2001) conducted a similar study in adolescents with TTH and migraines, applying PRT to two different headache groups (R1). Therefore, in the Study Design section, we have added further clarifications to improve understanding of our design between lines 128-131**.** Additionally, We also rearranged the title to "The Comparative Effectiveness of Progressive Relaxation Training on Pain Characteristics, Attack Frequency, Activity Self-Efficacy, and Pain-Related Disability in Women with Episodic Tension-Type Headache and Migraine" to match the changes we made to the method. Furthermore, we have revised thsample size (between lines 223-231) in the statistical analysis section for clarity. We hope that these changes will meet your expectations. These changes have also been reflected in the abstract to ensure consistency.

However, if you have a further suggestion, we will revise the manuscript in accordance with your suggestion.

Added Text in Study Design Section:"This study is prospective, experimental between-group study with premeasurements and postmeasurements. In this study, TTH and migraines participated in PRT sessions twice a week for six weeks. The aim of this study was to evaluate and compare the outcomes of these two distinct groups after the training.

Added text in Statistical analysis Section: The G* Power software (G* Power, Version 3.1.9.7 Franz Faul, Universität Kiel, Germany) was used to determine the sample size. The preliminary hypothesis was defined as difference in VAS scores over time (pre-test and post-test) and between groups. Accordingly, it was assumed that the time and group effects would have a moderate effect size in our study. With a Type I error rate of 𝛼=0.05 α=0.05 (95% confidence level) and a desired power of 1−𝛽=0.80 1−β=0.80, the required sample size for statistical analyses was calculated as 20 (R2).

Post-hoc power analysis was conducted after the study to evaluate whether the sample size used was sufficient. The post-hoc power analysis revealed that the power of the study was 98%. This indicates that the sample size used in the study was statistically sufficient

R1: Fichtel Å, Larsson B. Does relaxation treatment have differential effects on migraine and tension‐type headache in adolescents?. Headache. 2001;41(3): 290-296. doi: 10.1046/j.1526-4610.2001.111006290.x

R2: Faul F, Erdfelder E, Buchner A, Lang AG. 2009. Statistical power analyses using G* Power 3.1: Tests for correlation and regression analyses. Behavior Res Met, 41: 1149-1160)

- The Randomization

Since the study included two different headache groups, randomization was not performed. This study is a prospective, experimental between-group study with premeasurements and postmeasurements.

- The Blinding

In this study, to minimize potential bias, the statistician responsible for analyzing the data was blinded to the group allocations throughout the study. However, the lack of blinding for the evaluators and participants regarding the study group represents a limitation. This has been addressed in the limitations section of the manuscript
